# From body shaping to character formation: international research progress and mechanisms of exercise training interventions for juvenile delinquency

**DOI:** 10.3389/fpsyg.2026.1888577

**Published:** 2026-09-09

**Authors:** Jing Xu, Hongjin Chen, Mengdi Tian

**Affiliations:** 1 Fujian Police College Department of Police Tactics, Fujian, China; 2 College of Physical Education Sichuan Agricultural University, Ya’an, China; 3 School of Physical Education and Health, Sichuan Technology and Business University, Meishan, China

**Keywords:** exercise training, international research, intervention, juvenile delinquency, positive youth development, psychosocial mechanisms

## Abstract

Juvenile delinquency is a global public health concern requiring effective prevention strategies. This narrative review summarizes and synthesizes international evidence on structured exercise and sport-based interventions for juvenile delinquency, examining theoretical frameworks, mechanisms, program characteristics, and effectiveness. We conducted a literature search in PubMed and Web of Science; evidence indicates that intervention effectiveness depends critically on program design and implementation quality—long-term structured programs with trained coaches produce the most robust effects. Exercise facilitates behavioral change through interconnected physiological, psychological, and social mechanisms, with integration of cognitive-behavioral therapy and family support yielding stronger outcomes than exercise alone. Structured exercise shows promise as a developmental intervention for delinquency prevention, but success requires theory-driven design, skilled mentorship, and cultural sensitivity. Future research should prioritize rigorous longitudinal designs and cultural adaptation in underrepresented regions.

## 1 Introduction

Juvenile delinquency is a global public health and social problem. It not only causes serious harm to individual development, including interruption of education, barriers to employment, and mental-health problems, but also imposes substantial economic and safety burdens on society (Her et al., 2025). Traditional judicial responses such as incarceration and punishment mainly focus on post-event control. Their effectiveness in preventing recidivism and promoting individual development is often limited, and such responses may also produce negative consequences such as stigma (Junewicz and Billick, 2021). Accordingly, scholars and practitioners have increasingly turned to developmental and preventive intervention approaches that seek to reduce risk factors and strengthen protective factors. In this context, exercise training has been widely regarded as a promising intervention strategy because it is non-stigmatizing, highly participatory, and capable of promoting prosocial behavior while reducing antisocial and delinquent behavior through multiple pathways (Shenoi et al., 2022).

Across North America, Europe, Asia, and Oceania, a growing number of studies and practical programs have attempted to prevent juvenile delinquency through sport and exercise (Ramer et al., 2025). Beyond these well-studied regions, emerging research from North Africa has also contributed valuable insights into adolescent physical activity patterns and their behavioral correlates. A cross-sectional study of 669 Moroccan adolescents aged 14–19 years found that 38% did not meet the recommended duration of daily moderate-intensity physical activity, with boys generally more active than girls, while 36% reported watching television for more than 2 h per day and 42% used computers for a similar duration (El Oirdi et al., 2021). Another investigation among Moroccan high school students aged 15–20 years reported that 74.2% of boys and 46.9% of girls were physically active, yet a substantial proportion—45.5% of girls and 20% of boys—spent more than 2 h daily watching television, and a statistically significant negative correlation was found between physical activity levels and sedentary behaviors (El Oirdi et al., 2023). These findings underscore the high prevalence of physical inactivity and sedentary lifestyles in North African adolescent populations and highlight the importance of understanding physical activity patterns in non-Western contexts. Such evidence is particularly relevant when considering culturally adapted exercise interventions for regions where sedentary behaviors and associated health risks are prevalent, and it reinforces the broader applicability of physical activity research to adolescent behavioral health across diverse cultural and socioeconomic settings. Although these programs are grounded in different theoretical frameworks, they share the assumption that structured exercise can provide alternative activities, cultivate self-discipline, build positive social relationships, and shape character. Positive youth development theory emphasizes the cultivation of competence, confidence, character, connection, and caring through meaningful participation opportunities. Social learning theory suggests that adolescents can learn cooperation, rule respect, and emotion regulation by observing and imitating prosocial models such as coaches and teammates in sport teams. Strain theory proposes that exercise provides a legitimate channel for releasing stress, frustration, and tension, thereby reducing the likelihood that adolescents will cope with negative emotions through antisocial behavior (Pechorro et al., 2022). Together, these theories form multiple pathways through which exercise may intervene in juvenile delinquency (Boering et al., 2024).

A substantial body of evidence supports the positive role of exercise training in reducing adolescent problem behaviors. A study of adolescents visiting pediatric emergency departments found that participation in moderate-to-vigorous physical activity and sports was significantly associated with a lower risk of depression (Shenoi et al., 2022). In contrast, excessive screen time (at least 3 h/day) was associated with increased risks of conduct disorder, depression, and substance use (Shenoi et al., 2022). These findings suggest that increasing active physical activity while reducing sedentary behavior may help improve behavioral and emotional health. Exercise programs may be especially important for adolescents with attention-deficit/hyperactivity disorder (ADHD) and disruptive behavior disorders. However, one study of African American children found that children with these conditions had lower participation in sports (12%) and physically active after-school programs (31%) than their peers, while participation in sedentary after-school programs was higher (54%) (Ramer et al., 2025). Such participation inequality highlights the need to create inclusive exercise opportunities for high-risk youth. In addition, research comparing organized and non-organized physical activity among adolescents with disabilities and/or behavioral disorders showed that organized physical activity was more effective in promoting school integration and motor-skill development, leading to more positive psychosocial outcomes (Alexandru et al., 2020).

Despite these prospects, exercise-based intervention for juvenile delinquency still faces many challenges. First, intervention effects may be moderated by individual differences, such as conduct-disorder subtypes (childhood-onset versus adolescence-onset) and the presence of callous-unemotional traits, which may involve different neurobiological foundations and behavioral manifestations (Dugré et al., 2026; Gao and Staginnus, 2024). Second, how to reach and continuously engage the highest-risk youth remains a practical difficulty. Family environment, peer relationships, and socioeconomic status also profoundly influence intervention outcomes (LoBraico et al., 2020; Goetz et al., 2025). Future research and practice should therefore identify target groups more precisely, design theory-driven and evidence-based multimodal exercise interventions, and establish collaborative support models linking families, schools, community service agencies, and sport professionals, thereby maximizing the potential of exercise in preventing juvenile delinquency and promoting positive development (Ramer et al., 2025).

To ensure conceptual clarity and establish the scope of this review, it is necessary to distinguish three related but non-interchangeable intervention approaches: exercise training interventions, sport-based interventions, and multicomponent youth programs that include a physical activity component. Exercise training typically refers to a structured, repetitive, and purposeful physical activity regimen aimed at improving or maintaining one or more components of physical fitness. Sport-based interventions, in contrast, are organized, rule-governed, and often competitive activities that inherently involve social interaction, teamwork, and adherence to norms, thereby providing a natural context for psychosocial development. Multicomponent programs, on the other hand, incorporate physical activity as one of several complementary elements (e.g., alongside academic tutoring, vocational training, or psychological counseling), but the physical activity itself may not be the primary therapeutic or developmental vehicle. The present review focuses specifically on structured exercise and sport-based interventions in which physical activity serves as the central or primary intervention medium, rather than merely an adjunct component. This focus is justified by two considerations. First, sport and exercise provide a unique combination of physiological stimulation, skill mastery, and social interaction that is not fully replicated in other program components. Second, a growing body of intervention research has evaluated sport and exercise as standalone or principal strategies for delinquency prevention and youth development, making a targeted synthesis both feasible and necessary. Programs in which physical activity is only a minor or optional addition to a predominantly academic, vocational, or psychotherapeutic curriculum are excluded from this review, although their integrated effects are discussed in Section 8 as part of synergistic strategies. By delineating this boundary, the review aims to provide a focused and actionable synthesis for researchers, practitioners, and policymakers interested in the specific contribution of exercise and sport to juvenile delinquency intervention.

Based on the above scoping, the present review addresses the following research questions: (1) What are the theoretical frameworks that underpin the use of exercise and sport interventions for juvenile delinquency prevention and intervention? (2) Through what physiological, psychological, and social mechanisms do exercise and sport interventions potentially reduce delinquent behavior and promote positive youth development? (3) What are the key types, design characteristics, and implementation features of existing international exercise-based intervention programs for juvenile delinquency? (4) What is the empirical evidence regarding the effectiveness of these interventions, and what moderators influence their outcomes? (5) What are the current challenges, limitations, and future directions for research and practice in this field?

The overarching objective of this review is to synthesize international empirical evidence on exercise and sport-based interventions for juvenile delinquency, with the aim of providing researchers, practitioners, and policymakers with a comprehensive and actionable evidence base to inform the design, implementation, and evaluation of such programs.

## 2 Theoretical perspectives on juvenile delinquency and the fit with exercise intervention

### 2.1 Implications of mainstream criminological theories for exercise intervention

Strain theory provides a key perspective for understanding juvenile delinquency. It argues that when individuals perceive a large gap between socially valued goals, such as success and status, and their ability to achieve those goals through legitimate means, frustration and pressure arise. This state of strain may push individuals toward crime or other deviant behaviors as alternative ways of obtaining satisfaction or releasing emotions (Galinari and Bazon, 2021). Exercise training, as a structured and socially recognized activity, provides adolescents with a legitimate pathway to achievement. By mastering sport skills, winning competitions, or reaching personal fitness goals, adolescents can gain a sense of achievement and self-efficacy in the sport domain, thereby alleviating strain and frustration arising from blocked social goals (Cardona-Isaza et al., 2025). Participation in prosocial activities such as sport has been shown to enhance life satisfaction and positive emotional experiences, which may counteract negative emotions and reduce the risk of strain-induced delinquency (Cardona-Isaza et al., 2025). Thus, exercise intervention can be regarded as a buffering mechanism that directly addresses the causes of delinquency highlighted by strain theory by providing alternative routes to success and positive emotional outlets.

Social control theory emphasizes that the strength of bonds between individuals and society is central to crime prevention. These bonds include attachment to conventional institutions such as family and school, involvement in routine activities, commitment to mainstream values, and belief in social norms (Higgins et al., 2020). When these bonds are weak or broken, the likelihood of offending increases. Within this framework, sport teams function as micro-social systems that allow adolescents to develop close and positive relationships with prosocial adults, such as coaches, and with peers who share similar goals (Mikytuck and Woolard, 2021). Coaches are not only skill instructors but may also become role models and sources of emotional support, while teammate relationships can cultivate belonging and collective responsibility. Research on serious adolescent offenders has shown that support from friends, especially non-deviant peers, has a protective effect on positive development (Mikytuck and Woolard, 2021). By strengthening these prosocial bonds, sport participation increases adolescents’ involvement in conventional activities and adherence to team norms, thereby enhancing social control and reducing the likelihood of disengagement from social constraints and engagement in criminal behavior (Dunlop et al., 2024).

Social learning theory states that behavior, including criminal behavior, is learned through observation, imitation, and reinforcement. The environment in which adolescents live, especially the models and peers within it, has a profound influence on behavioral formation (Filkin et al., 2022). In sport settings, coaches and outstanding teammates can serve as powerful models of prosocial behavior. Through daily training and competition, coaches not only teach sport skills but also demonstrate and reinforce values and behavioral norms such as respect for rules, fair play, teamwork, emotion management, and perseverance (Cardona-Isaza et al., 2025). This process is a form of vicarious reinforcement. When adolescents observe that rule-following and effort are rewarded with praise from coaches, recognition from teammates, or team success, they are more likely to internalize and imitate such behavior. Conversely, aggression, cheating, and other antisocial behaviors are punished through fouls, substitution, or sanctions. Well-structured sport programs can therefore provide opportunities to learn prosocial behavior patterns and reinforce them through explicit rewards and consequences, potentially counteracting delinquent behavior patterns learned in adverse environments.

### 2.2 The role of exercise within the positive youth development framework

**Figure 1:**
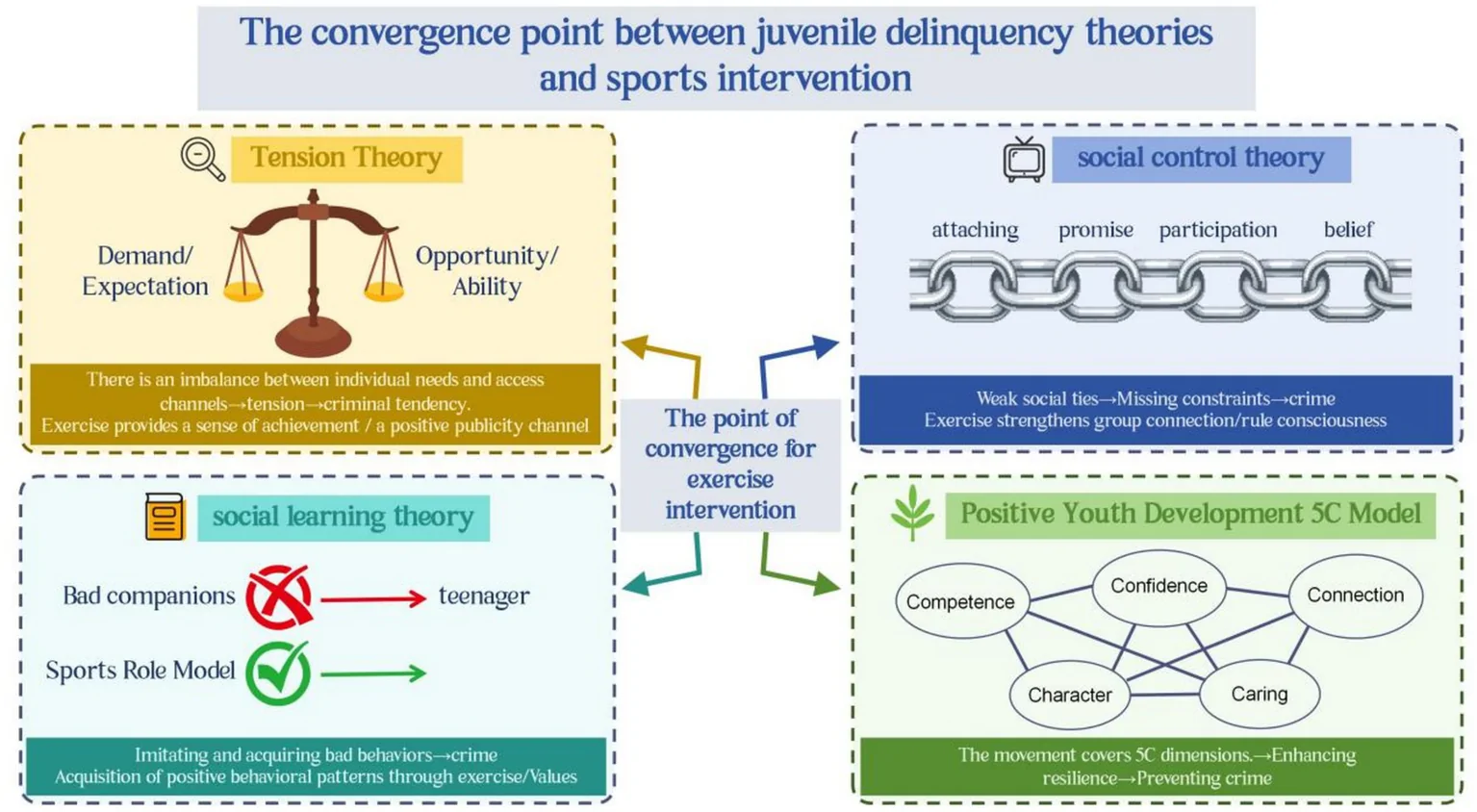
Convergence between juvenile-delinquency theories and sports intervention. The figure illustrates the alignment of strain theory, social control theory, social learning theory, and the 5C model of positive youth development with sports intervention, and shows how exercise can enhance adolescents’ comprehensive capacities and thereby help prevent and intervene in juvenile delinquency.

The positive youth development framework views adolescents as resources with developmental potential rather than as problems to be corrected. Its core objective is to cultivate the five characteristics of competence, confidence, character, connection, and caring, commonly referred to as the 5C model (Arslan et al., 2025). These characteristics constitute key protective factors against delinquency. Research has shown that juvenile delinquency is closely associated with risk factors such as incomplete family structure, insufficient educational support, and a lack of positive social connections (Arslan et al., 2025; Çağlar, 2025). Therefore, delinquency-prevention interventions should proactively build developmental assets rather than merely manage risk or fill time. Structured exercise training provides an ideal context for this aim. Sport-leadership programs in juvenile correctional institutions provide incarcerated youth with crucial opportunities for moderate-to-vigorous physical activity (Wahl-Alexander and Jacobs, 2022a). More importantly, skill practice and teamwork directly promote the 5C characteristics. Participants learn sport skills, which improves competence; they take roles and complete tasks in teams, which builds confidence; they follow rules and respect opponents and teammates, which shapes character; and shared training and competition foster connection and caring (Wahl-Alexander and Jacobs, 2022b). This approach, which builds developmental assets through exercise, goes beyond simple physical training. Studies have confirmed that adolescents participating in such programs show more prosocial interactions and fewer antisocial behaviors, and that activity levels become more consistent as participation increases, suggesting stronger group integration (Wahl-Alexander and Jacobs, 2022b; Wahl-Alexander et al., 2023). These findings indicate that well-designed sport programs can embed positive youth development principles and produce lasting positive effects on cognition, emotion, and social development, providing a deeper solution to delinquency prevention than traditional punishment or skills-only training (Wills, 2025) (Figure 1).

## 3 Mechanisms by which exercise training influences juvenile delinquency

### 3.1 Physiological and neuropsychological mechanisms

The physiological and neuropsychological mechanisms through which regular exercise may intervene in juvenile delinquency mainly involve positive regulation of neurotransmitter systems, brain structure and function, and stress-response systems. Exercise can regulate neurotransmitters closely related to emotion and behavioral control. Juvenile offenders are often characterized by emotional dysregulation, aggression, and impulsivity (Franco-O’Byrne et al., 2021). Regular physical activity has been shown to increase levels of serotonin and endorphins in the brain. These substances are important for mood stability, pleasure, and pain reduction. Increased serotonin may improve mood and reduce depressive and anxiety symptoms, while endorphins can produce a natural sense of wellbeing. Together, these effects may reduce the tendency to seek stimulation or discharge negative emotions through aggression or deviant behavior (Stutts and Cohen, 2022). Improvement in the neurochemical environment therefore directly targets emotional instability and weak impulse control, two core risk factors commonly observed in juvenile delinquency (Jian et al., 2026).

Exercise also promotes the development and functioning of the prefrontal cortex, a key brain region responsible for higher-order executive functions. Executive functions include impulse control, decision-making, planning, and emotion regulation, and deficits in these capacities are strongly associated with juvenile delinquency (Loureiro et al., 2023). Neuroimaging studies indicate that juvenile offenders may show reduced gray-matter volume or abnormal functional connectivity in regions involved in social–emotional regulation and theory of mind, including the prefrontal cortex, temporal lobe, and parietal lobe (Franco-O’Byrne et al., 2021). Regular exercise can stimulate neuroplasticity in these regions, promote neuronal growth and synaptic connections, and enhance executive function. By improving prefrontal-cortex function, exercise may directly strengthen adolescents’ ability to inhibit impulsive behavior, enabling more rational and socially appropriate decisions when facing conflict or temptation rather than resorting to violence or illegal behavior (Anderson et al., 2024).

In addition, exercise helps adolescents cope with daily stress by improving sleep quality and lowering physiological stress responses, thereby reducing deviant behavior triggered by stress. Many justice-involved youth have high levels of trauma exposure and post-traumatic stress symptoms, which can lead to chronic physiological stress and dysregulated stress hormones such as cortisol (Modrowski et al., 2021; Zaverdinou et al., 2026). Regular exercise is an effective non-pharmacological stress-management strategy. It can help regulate the hypothalamic–pituitary–adrenal axis and lower baseline cortisol levels, creating a more balanced stress-response system. Exercise habits also improve sleep structure and quality, while sleep deprivation itself can impair prefrontal functioning and exacerbate impulsivity and emotional problems (Gaylord-Harden et al., 2020). By reducing physiological arousal and improving sleep, exercise provides a stable mind–body foundation that allows adolescents to cope with academic, family, and peer stress in healthier ways rather than turning to substance use, violence, or other criminal activities (Filkin et al., 2022).

### 3.2 Psychological and social-cognitive mechanisms

A core mechanism of exercise-based intervention lies in the reshaping of psychological and social-cognitive functions. Juvenile offenders commonly show low self-efficacy, difficulties in emotion regulation, weak impulse control, and social-cognitive biases (Franco-O’Byrne et al., 2021; Loureiro et al., 2023). Exercise training provides structured successful experiences that can improve these psychosocial deficits. In sport contexts, adolescents repeatedly practice, face challenges, master skills, and achieve measurable success. Such mastery experiences are among the strongest sources of self-efficacy. When adolescents believe that their own effort can help them achieve sport goals, this belief may generalize to other life domains, reducing their tendency to seek recognition or prove self-worth through deviant behaviors such as violence or theft (Cardona-Isaza et al., 2025). A study of adolescent offenders in Spain showed that an intervention program enhancing self-efficacy effectively reduced relapse in substance use, indirectly supporting the importance of strengthening perceived self-control in reducing problem behavior (Fernández-Moreno et al., 2024). Teamwork and positive coach feedback can also enhance adolescents’ overall self-worth and compensate for self-esteem damaged by academic failure, family problems, or peer exclusion (Higgins et al., 2020).

Exercise requires participants to set and pursue both short-term and long-term goals, a process central to cultivating delayed gratification and perseverance. From learning a new movement to winning a competition, adolescents must invest continuous effort and overcome boredom, fatigue, and frustration. This experience directly trains willpower and resilience. Juvenile offenders often display irrational decision-making styles, such as excessive reliance on intuition or hypervigilance in high-risk situations, and they may lack careful consideration of long-term consequences (Cardona-Isaza et al., 2025; Loureiro et al., 2023). The sport-training cycle of goal setting, planning, execution, evaluation, and adjustment is essentially training in rational decision-making and executive function. When adolescents transfer persistence and goal-oriented skills to academics, career planning, or interpersonal conflict resolution, they may be more likely to choose constructive, socially acceptable strategies rather than immediate but harmful responses such as violence or crime. Research in Chile found that weak family supervision, deviant peer relationships, and poor management of leisure time were important risk factors for the transition from child welfare to juvenile justice systems (Zambrano et al., 2023). Exercise training can counter these risks by providing structured leisure activities, positive peer models, and adult supervision.

Competitive sport settings naturally involve emotional challenges such as defeat, fouls, and conflicts with teammates or opponents, providing real-time practice for emotion regulation and impulse control. Juvenile offenders often show abnormal emotional processing, including attentional bias toward threatening stimuli and reduced experience of social emotions such as envy and schadenfreude, which are linked to dysfunction in prefrontal and temporoparietal regions (Franco-O’Byrne et al., 2021; Pino et al., 2023). In sport, intense emotions such as anger, frustration, and anxiety emerge immediately. Coaches and team environments serve as external regulators and teachers at these moments. Effective coaches guide adolescents to identify emotions, manage them through breathing, positive self-talk, and attentional shifting, and transform frustration into motivation for improvement rather than aggressive behavior. Learning to remain calm and follow rules under pressure, even when disagreeing with a referee’s decision, directly trains prefrontal inhibition and cognitive reappraisal. Because poor control of impulsivity and aggression is associated with violent offending, repeated practice in sport may help adolescents internalize regulatory strategies and respond more adaptively to provocation, pressure, or injustice in daily life (Ashton et al., 2024).

### 3.3 Social relationships and social capital mechanisms

Building positive adult connections is a key social mechanism through which exercise training can intervene in juvenile delinquency. Coaches, as non-parental authority figures, play an important role in adolescent development. Many adolescents with behavioral problems lack adequate family support, and coaches can provide stable and positive adult relationships that fill this gap. Such relationships involve not only skill guidance but also emotional support, behavioral monitoring, and value guidance, helping to form protective attachments. Juvenile delinquency is closely related to family-environment risks such as parental divorce (van de Weijer and Kroese, 2025) and childhood trauma (Alexander and Kerig, 2025). A trustworthy coach can therefore become an alternative positive model who helps adolescents build trust in and respect for authority through daily interactions and rule setting, reducing the risk of deviant behavior (Tian et al., 2025).

Reshaping peer networks is another core social process. Participation in prosocial sport teams can fundamentally alter adolescents’ social environments, helping them move away from deviant peer groups and into networks governed by positive behavioral norms. Peer influence is a major predictor of juvenile delinquency. For example, homophily with drug-using peers significantly increases later drug-use variety among adolescent offenders (Drozdova et al., 2022), and association with deviant peers is an important risk factor for offending (Guo, 2025). When adolescents join sport teams centered on sportsmanship and teamwork, their daily interaction partners shift from peers who may encourage antisocial behavior to teammates who encourage cooperation, discipline, and achievement. This change reduces opportunities for crime and negative social learning and also reshapes social identity. Adolescents may begin to see themselves as athletes rather than troublemakers, internalizing behavioral standards consistent with this new identity.

**Figure 2:**
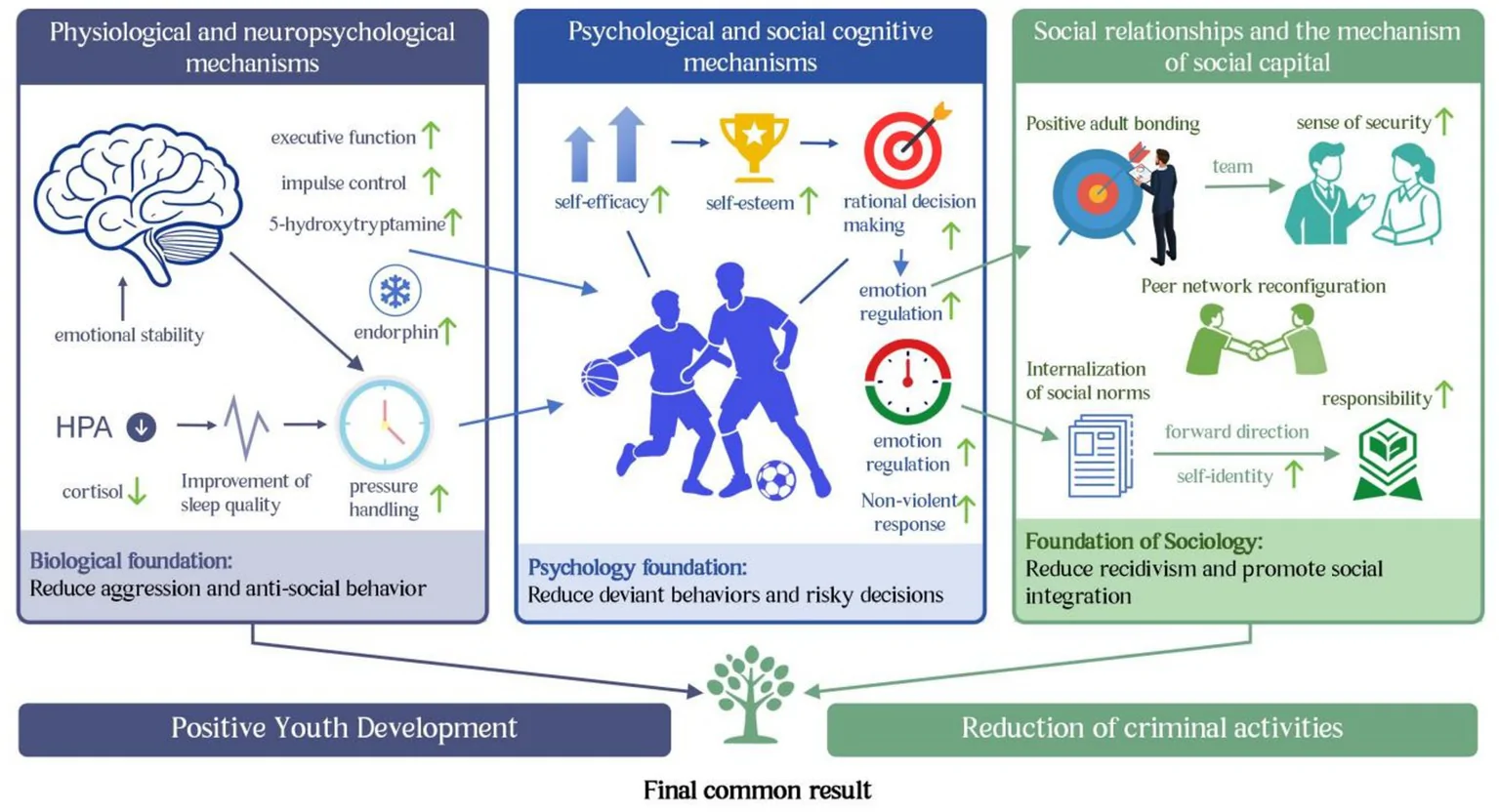
Mechanisms linking exercise intervention to positive youth development and reduced criminal behavior. The figure summarizes physiological and neuropsychological mechanisms, psychological and social-cognitive mechanisms, and social-relationship/social-capital mechanisms.

Enhancing social integration and internalizing norms are direct ways in which team sports promote prosocial development. A sport team is a micro-social system that requires participants to follow clear and non-negotiable rules and respect the decisions of authorities such as referees. On the field, rule violations immediately lead to clear consequences, such as fouls, warnings, or removal from play. This directly reinforces the relationship between behavior and consequence and helps adolescents understand the importance of rules. Juvenile offenders often show insufficient internalization of social norms, such as higher moral disengagement or deficits in theory of mind for negative emotional states (Loureiro et al., 2023; Agudelo Rico et al., 2024). Through repeated participation in rule-governed team sports, adolescents can practice delayed gratification, impulse control, and consideration of collective interests. Successful team integration and the collective honor and personal achievement associated with norm adherence can also increase life satisfaction and positive emotional experiences (Cardona-Isaza et al., 2025), further consolidating prosocial behavior and reducing the risk that frustration or alienation leads to delinquency (Figure 2).

## 4 Types and design characteristics of international exercise-intervention programs

### 4.1 Community-based preventive programs

Community-based preventive programs are an important international strategy for intervening in juvenile delinquency. They target adolescents in the community, especially those in high-risk neighborhoods, by providing safe and organized sport activities that occupy leisure time and reduce exposure to criminal opportunities and deviant behavior. These programs emphasize accessibility and attractiveness, often operating free of charge or at low cost and intentionally being scheduled during high-crime periods such as evenings and weekends. A classic example is the Midnight Basketball program in the United States, which attracted adolescents into safe community settings by providing structured basketball activities late at night (McDonough and Knight, 2023). Successful implementation depends on stable venues, trained leadership, and the natural integration of life-skills training into sport. When physical activity is combined with life-skills education, it may more effectively promote prosocial behavior and self-efficacy, contributing to the prevention of substance use and delinquency (Nelson et al., 2022). For example, one study of justice-involved youth found that participation in extracurricular activities, including sports, significantly reduced the risk of opioid misuse, although protective effects differed across racial and ethnic groups (Rigg and Johnson, 2022). Community programs must therefore consider the cultural and socioeconomic background of target populations to ensure inclusiveness and effectiveness (Mao, 2025).

The theoretical basis of such programs is to change adolescents’ daily behavior patterns and social networks by providing structured alternatives. When adolescents invest time and energy in supervised sport activities, their contact with deviant peers and criminal settings decreases. Teamwork, rule compliance, and coping with frustration in sport also function as implicit character education and life-skills training. Evidence suggests that integrating cognitive-behavioral principles into sport interventions can reduce juvenile delinquency. A meta-analysis from the Netherlands found that sport-based interventions combined with responsive cognitive-behavioral therapy, such as Only You Decide who you Are, showed positive effects in preventing delinquency (Meulen et al., 2026). Sustainability and leadership quality are also crucial. Trained coaches and instructors ensure safety and serve as positive models who build trust with adolescents and guide them toward positive values and goals. Community-level collaboration is equally important. In Victoria, Australia, the Communities That Care program significantly reduced municipal youth crime rates from 2010 to 2019, especially property and fraud offenses among youth aged 10–17 years (Rowland et al., 2022). This suggests that embedding sport interventions within broader community support networks may produce more durable preventive effects. Challenges remain, including stable funding, equitable access for girls and minority groups, and accurate assessment of long-term behavior change (McDonough and Knight, 2023).

### 4.2 Corrective programs for youth in the justice system

For adolescents who have been convicted or are on probation or supervision, incorporating exercise into correctional plans is an important area of international judicial practice. These programs are often embedded in juvenile detention centers or community-correction systems and use structured sport as an intervention medium. A national cross-sectional survey of long-term secure juvenile correctional facilities in the United States found that more than half (55.1%) operated sport programs (McDonough and Knight, 2023). Such programs provide incarcerated youth with opportunities for health promotion and positive development to counter the adverse health effects of confinement (McDonough and Knight, 2023). However, access is unequal; girls are generally offered fewer opportunities than boys, highlighting the challenge of ensuring equitable sport participation within the justice system (McDonough and Knight, 2023). Corrective exercise programs usually emphasize discipline, responsibility reconstruction, and explicit behavior-consequence links. Participation may be tied to behavioral performance, with misconduct leading to loss of participation privileges. This design simulates social rules and helps adolescents understand the relationship between action and consequence. Given that 73–86% of justice-involved youth may be diagnosed with mental disorders during adolescence or adulthood, correctional programs must go beyond punishment and integrate developmental support (Seker et al., 2022). Innovative approaches such as juvenile-justice-based interdisciplinary collective care link justice systems with community organizations to address both mental-health needs and criminogenic risk factors (Brown and Perez, 2024).

The central challenge is how to balance punishment and development and maintain effects after youth return to the community. Purely punitive environments may exacerbate trauma. Approximately 80% of youth entering probation report at least one traumatic experience, and trauma, suicidality, and mental-health problems have increased over time among youth entering juvenile justice systems (Kim et al., 2021). Trauma exposure is indirectly associated with offending through post-traumatic dissociation and reckless or self-destructive behavior (Modrowski et al., 2021). Program design should therefore adopt a trauma-informed approach and avoid secondary harm. Trauma-informed juvenile justice requires meeting basic needs, changing narratives and underlying assumptions, and creating sustained systemic change (Moreland and Ressler, 2021). Second, resources available within facilities are limited, and structural barriers can reduce access to probation and community services (Fountain and Mahmoudi, 2021). Third, many youths experience disconnection after release; once intensive supervision ends, they may return to the same risk environments, including deviant peers, family dysfunction, and unsafe leisure time (Zambrano et al., 2023). Corrective sport programs therefore need transition mechanisms that connect institutional sport participation with community clubs, schools, and mentors so that positive identity and supportive relationships continue after release. Adult criminal outcomes are associated with juvenile justice involvement, indicating that correctional sport interventions should be integrated with long-term social support rather than treated as short-term activities (Copeland et al., 2023; Winn et al., 2025).

### 4.3 School-based integrated programs

Schools are the main everyday settings for adolescents and therefore key sites for early intervention and delinquency prevention. School-based integrated programs incorporate exercise interventions and positive development strategies into daily education systems or after-school activities, allowing early identification and guidance of students who show signs of behavioral risk. Their advantage lies in the use of existing infrastructure and professional networks, including physical-education teachers and counselors, who interact frequently with students and can observe behavioral changes and potential risks (Higgins et al., 2020). School bonding and commitment are closely associated with offending trajectories (Higgins et al., 2020). For students with academic difficulty, weak school attachment, or emotional and behavioral problems, developmental physical education and after-school sport programs can provide channels for rebuilding connections with school. Compared with justice-system interventions, school-based programs are earlier, less stigmatizing, and more capable of embedding prevention into everyday development.

**Figure 3:**
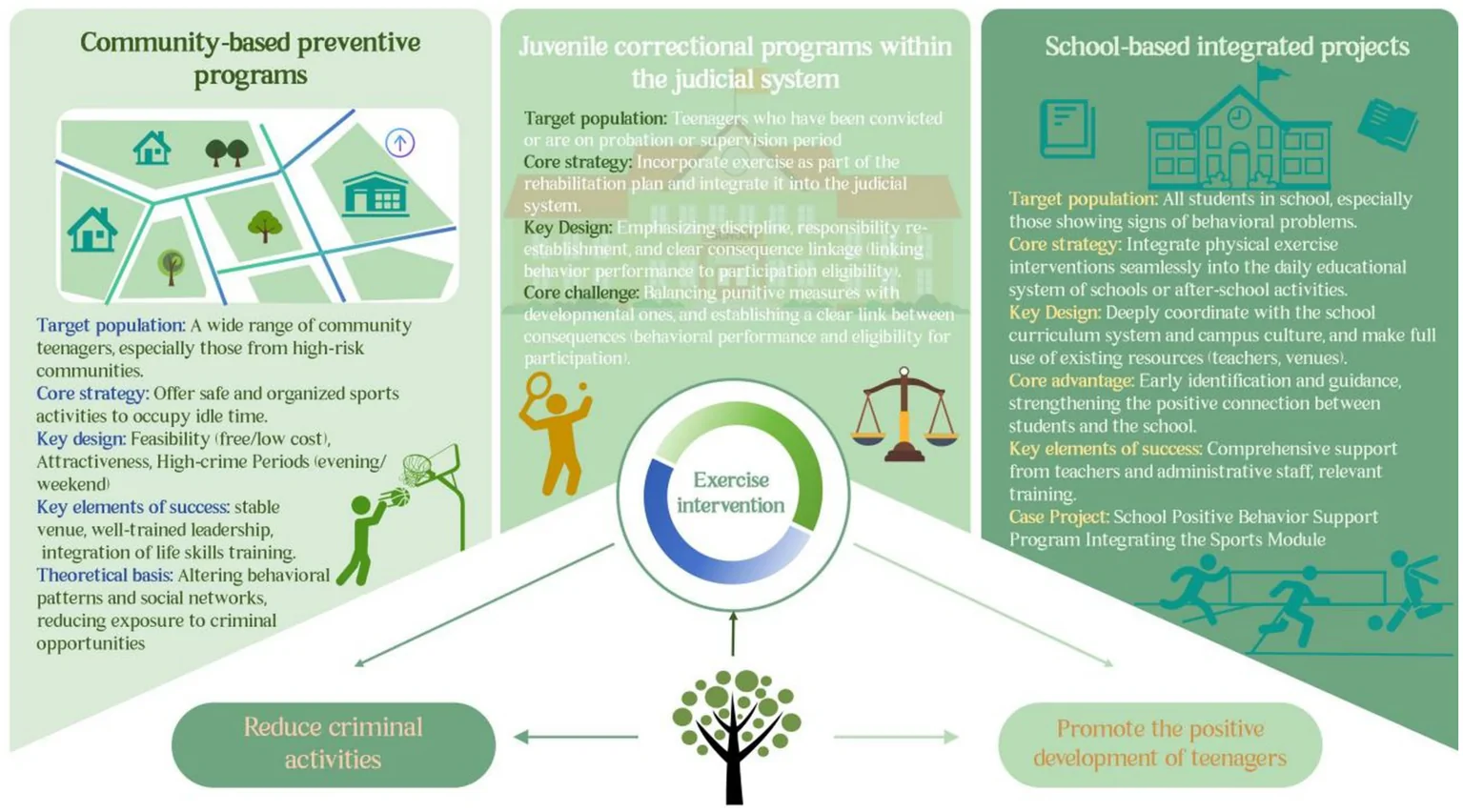
Types and design characteristics of exercise-intervention models. The figure introduces target populations, core strategies, and design features of community preventive programs, judicial corrective programs, and school-based integrated programs, and clarifies how these models reduce juvenile delinquency through different pathways.

Successful implementation depends on deep coordination between program design, existing curricula, and school culture. Sport modules should not be isolated add-ons but should complement academic education and character education. For example, team sports can be combined with conflict-resolution courses, and physical education can embed training in emotion management and self-regulation. Full support from teachers and administrators is essential. Teachers, especially physical-education teachers, need to understand that their role is not only to teach sport skills but also to guide social–emotional learning and positive behavior. From a broader international perspective, integrated community- and school-based prevention models have shown promise. The Communities That Care program in Australia, although a broader community model, relies partly on coordinated efforts across systems including schools and has been associated with reduced youth crime rates (Rowland et al., 2022). This suggests that school-based sport interventions should be embedded within multisystem collaboration involving families, social services, and community sport organizations (Figure 3).

## 5 Evaluation of international empirical evidence on the effectiveness of exercise intervention

To provide a transparent and structured overview of the empirical evidence base, this section begins with a summary table (Table 1) presenting the key characteristics of the included studies. This table is organized to facilitate cross-study comparison across geographic regions, study designs, intervention types, and outcome domains. Following the table, findings are narratively synthesized by region—North America, Europe, Asia and other regions—with particular attention to program effectiveness, methodological quality, and contextual factors that may influence intervention outcomes.

**Table 1:** Summary of included empirical studies on exercise and sport-based interventions for juvenile delinquency.

First author and year	Country/context	Study design	Type of intervention	Duration/frequency	Outcomes assessed	Main findings
Shenoi et al. (2022)	USA (Pediatric ED)	Cross-sectional survey	Self-reported MVPA and sports participation	Not specified (cross-sectional)	Depression, conduct disorder, substance use	MVPA and sports participation significantly associated with lower depression risk; excessive screen time (≥3 h/day) linked to increased conduct disorder, depression, and substance use.
Ramer et al. (2025)	USA	Cross-sectional survey	After-school programs: sports, physically active, sedentary	Not specified	Participation rates in different program types	Children with ADHD/disruptive disorders had lower sports participation (12%) and physically active program participation (31%), but higher sedentary program participation (54%) than peers.
Alexandru et al. (2020)	Romania	Cross-sectional comparative	Organized vs. non-organized physical activity	Not specified	School integration, motor skills, psychosocial outcomes	Organized physical activity was more effective in promoting school integration and motor-skill development than non-organized activity.
Wahl-Alexander and Jacobs (2022a)	USA (Juvenile correctional facility)	Observational (pre-post)	Sport leadership program	Multiple sessions over program period	Physical activity levels (accelerometry)	Sport leadership program provided crucial opportunities for MVPA among incarcerated youth; activity levels became more consistent with increased participation.
Wahl-Alexander and Jacobs (2022b)	USA (Juvenile correctional facility)	Observational	Sport leadership program	Multiple sessions over program period	Physical activity levels, prosocial/antisocial behaviors	Participants showed more prosocial interactions and fewer antisocial behaviors during program sessions; positive behavioral trends associated with program engagement.
Wahl-Alexander et al. (2023)	USA (Juvenile correctional facility)	Pre-post intervention	Behavior modification strategies + physical activity	Structured sessions over program period	Physical activity levels	Behavior modification strategies significantly increased physical activity levels among incarcerated youth.
Franco-O’Byrne et al. (2021)	Colombia	Cross-sectional neuroimaging	N/A (observational comparing groups)	N/A	Gray matter volume, executive function, social emotions (envy/schadenfreude)	Adolescent offenders showed abnormalities in complex social emotions associated with reduced gray-matter volume in prefrontal-temporal regions involved in mentalizing and executive function.
Fernández-Moreno et al. (2024)	Spain	Quasi-experimental	Contingency management program (including behavioral reinforcement)	Not specified	Substance use relapse (urinalysis)	Program enhancing self-efficacy effectively reduced substance use relapse, supporting importance of perceived self-control.
Cardona-Isaza et al. (2025)	Colombia	Cross-sectional	N/A (observational)	N/A	Decision-making styles, life satisfaction, prosociality	Rational decision-making related to prosocial behavior through life satisfaction; irrational styles (hypervigilance) related to behavioral difficulties through negative emotions.
Rowland et al. (2022)	Australia (Victoria)	Quasi-experimental (community-level)	Communities That Care program (multicomponent community prevention, including sport components)	2010–2019	Municipal youth crime rates	Program significantly reduced property and fraud offenses among youth aged 10–17; effects sustained over decade.
McDonough and Knight (2023)	USA	National cross-sectional survey	Sport programs in correctional settings	N/A	Program availability, participation equity	55.1% of long-term secure facilities operated sport programs; girls offered fewer opportunities than boys; positive youth development cited as primary program purpose.
Her et al. (2025)	Taiwan	Cross-sectional clinical evaluation	N/A (observational)	N/A	Psychiatric disorders, academic achievement, offense type	89.76% of offenders had academic-achievement problems; academic failure significantly associated with offense type and specific psychiatric disorders.
Higgins et al. (2020)	Northern Ireland (Belfast)	Longitudinal cohort	N/A (observational examining school bonding)	Longitudinal	School bonding, offending trajectories	School bonding and commitment significantly associated with offending trajectories; sport participation may strengthen school attachment.
Mikytuck and Woolard (2021)	USA	Longitudinal	N/A (observational examining friendship support)	Longitudinal	Friendship support, positive development	Support from friends, especially non-deviant peers, had protective effect on positive development among serious adolescent offenders.
Guo (2025)	USA	Longitudinal (random-effects models)	N/A (observational)	Longitudinal	Self-control, morality, peer delinquency, offending	Self-control most protective when combined with strong moral values; exercise may strengthen self-control through persistence and delayed gratification.
Drozdova et al. (2022)	USA	Longitudinal	N/A (observational)	Longitudinal	Drug use homophily, drug-use variety	Homophily with drug-using peers significantly increased later drug-use variety among adolescent offenders.
Copeland et al. (2023)	USA	Longitudinal	N/A (observational)	Longitudinal (to adulthood)	Adult criminal outcomes	Juvenile justice involvement significantly associated with adult criminal outcomes, underscoring need for long-term intervention.
Walters (2021)	USA	Longitudinal	N/A (observational)	Longitudinal	Parental gambling, child gambling-delinquency relationship	Parental gambling significantly moderated child delinquency-gambling relationship, highlighting role modeling in home environment.
LoBraico et al. (2020)	USA	Longitudinal	N/A (observational)	Longitudinal	Family risk constellations, antisocial behavior	Specific constellations of family risk significantly predicted long-term adolescent antisocial behavior.

The following narrative synthesis organizes the empirical evidence by geographic region—North America, Europe, Asia and other regions—consistent with the study categorization presented in Table 1. Within each region, findings are discussed with attention to study design, program characteristics, outcome measures, and reported effect sizes. When available, findings from studies with stronger methodological quality (e.g., randomized controlled trials, quasi-experimental designs with control groups) are given greater weight in the synthesis. Observational studies are discussed primarily to identify correlates, risk factors, and contextual variables that may inform the design and targeting of future exercise interventions, rather than to establish causal effects (Luttenberger et al., 2022).

To improve the clarity and interpretability of the evidence synthesis, the following narrative is organized not only by geographic region but also by outcome type. Within each region, findings are grouped into four categories: (1) delinquency-related outcomes; (2) psychosocial outcomes; (3) educational outcomes; and (4) behavioral and health outcomes. This classification enables readers to discern the specific domains in which exercise and sport interventions demonstrate the strongest or most consistent evidence, while also highlighting areas where evidence remains limited or inconclusive.

### 5.1 Findings from North America

North America, particularly the United States, has accumulated extensive research evidence on exercise and sport-based interventions for juvenile delinquency. The following synthesis organizes this evidence by outcome type.

Delinquency-related outcomes. Randomized controlled trials and quasi-experimental studies indicate that high-quality, long-term, structured exercise programs can significantly reduce aggressive behavior, disciplinary records, and recidivism among participants (Meulen et al., 2026). Effective programs generally extend beyond physical training and integrate character education, skill training, and positive relationship building. For example, although the meta-analysis of the Only You Decide who you Are intervention came from Europe, its conclusion is consistent with the North American emphasis on structured and developmental sport intervention (Meulen et al., 2026). These effective programs typically include clear curricula that cultivate responsibility, respect, and decision-making, and they rely on trained adults to provide continuous support rather than merely organizing adolescents to play sports. Observational studies have further identified consistent risk factors—such as deviant peer associations, weak family supervision, and low school bonding—that exercise interventions may potentially address through the provision of structured prosocial alternatives.

Psychosocial outcomes. Research on psychosocial mechanisms has demonstrated that sport participation in North American correctional and community settings is associated with improvements in self-efficacy, emotion regulation, and impulse control (Cardona-Isaza et al., 2025; Franco-O’Byrne et al., 2021; Loureiro et al., 2023). A study of serious adolescent offenders found that support from friends, especially non-deviant peers, had a protective effect on positive development (Mikytuck and Woolard, 2021), suggesting that the peer environment cultivated in sport settings may enhance psychosocial adjustment. Neuroimaging research has revealed that adolescent offenders may show reduced gray-matter volume or abnormal functional connectivity in regions involved in social–emotional regulation (Franco-O’Byrne et al., 2021), and regular exercise may promote neuroplasticity in these regions, potentially strengthening executive functions and emotional control (Anderson et al., 2024). These psychosocial improvements are believed to mediate the relationship between sport participation and reduced antisocial behavior.

Educational outcomes. School bonding and commitment are closely associated with offending trajectories, and sport participation has been identified as a potential pathway for strengthening school attachment among at-risk youth (Higgins et al., 2020). For students with academic difficulty, weak school attachment, or emotional and behavioral problems, developmental physical education and after-school sport programs can provide channels for rebuilding connections with school. Studies have shown that participation in extracurricular activities, including sports, significantly reduces the risk of school disengagement, although protective effects may differ across racial and ethnic groups (Rigg and Johnson, 2022). The school-based sport context provides a structured environment where adolescents can experience success outside the academic domain, potentially buffering against the negative effects of academic failure on school commitment.

Behavioral and health outcomes. A study of adolescents visiting pediatric emergency departments found that participation in moderate-to-vigorous physical activity and sports was significantly associated with a lower risk of depression (Shenoi et al., 2022). In contrast, excessive screen time (at least 3 h/day) was associated with increased risks of conduct disorder, depression, and substance use (Shenoi et al., 2022). These findings suggest that increasing active physical activity while reducing sedentary behavior may help improve behavioral and emotional health. However, participation inequality remains a concern: one study of African American children with attention-deficit/hyperactivity disorder and disruptive behavior disorders found that these children had lower participation in sports (12%) and physically active after-school programs (31%) than their peers, while participation in sedentary after-school programs was higher (54%) (Ramer et al., 2025). Such disparities highlight the need to create inclusive exercise opportunities for high-risk youth. Regarding substance use, a study of justice-involved youth found that participation in extracurricular activities, including sports, significantly reduced the risk of opioid misuse, although protective effects differed across racial and ethnic groups (Rigg and Johnson, 2022).

### 5.2 Practices and evaluations in Europe

The United Kingdom, Germany, and other European countries have evaluated programs using football, boxing, and related sports for marginalized youth groups. The following synthesis organizes this evidence by outcome type.

Delinquency-related outcomes. European sport interventions often target socially marginalized adolescents with high delinquency risk and use highly structured team or individual sports to provide alternative channels for social participation (Filkin et al., 2022). These programs have shown positive effects in improving social behavior and reducing street disorder, with the core mechanism being the replacement of risky social networks and activity patterns with organized sport environments. A retrospective interview study of adult prisoners in England, for instance, showed that early educational alienation and association with antisocial peers were important pathways into offending (Filkin et al., 2022), and sport programs can counter these pathways by providing disciplined participation, adult guidance, and opportunities for belonging. A meta-analysis from the Netherlands found that sport-based interventions combined with responsive cognitive-behavioral therapy, such as Only You Decide who you Are, showed positive effects in preventing delinquency (Meulen et al., 2026).

Psychosocial outcomes. European studies generally attach importance to process evaluation and examine how the quality of coach-participant relationships mediates program effects. Such relationships go beyond skill instruction and resemble developmental mentorship (Cardona-Isaza et al., 2025). High-quality relationships characterized by trust, respect, consistency, and emotional support provide at-risk adolescents with a safe attachment object and positive behavior model, helping satisfy psychological needs such as belonging and competence. Nordic countries emphasize the concept of sport for all, with research focusing more on how sport participation promotes social inclusion and indirectly prevents crime through enhanced psychosocial integration (Higgins et al., 2020). This approach is consistent with developmental ecological perspectives, which emphasize the dynamic interaction between individual propensity and environmental influence—inclusive and supportive social environments may reduce population-level exposure to delinquency risk.

Educational outcomes. European research has consistently identified educational alienation and truancy as key pathways into offending (Filkin et al., 2022). Sport programs in school and community settings are designed to counteract these pathways by providing structured engagement that can re-engage marginalized youth with educational institutions. The integration of sport with educational support has shown promise in improving school attendance and reducing dropout rates among at-risk populations, though rigorous evaluation of these combined effects remains limited.

Behavioral and health outcomes. While less prominently featured in the European delinquency-intervention literature, behavioral outcomes such as substance use reduction have been evaluated in some program contexts. Research from Spain, for example, showed that an intervention program enhancing self-efficacy effectively reduced relapse in substance use among adolescent offenders, indirectly supporting the importance of strengthening perceived self-control in reducing problem behavior (Fernández-Moreno et al., 2024). This finding suggests that sport-based programs, when combined with cognitive-behavioral approaches, may positively influence substance-use behaviors through enhanced self-regulatory capacity.

### 5.3 Exploration in Asia and other regions

In Asia, countries and regions such as Japan and Singapore have paid particular attention to the unique role of school sport clubs in character formation. Through strict training, rule compliance, and teamwork, these activities cultivate discipline, responsibility, and collective belonging. The following synthesis organizes the available evidence by outcome type.

Delinquency-related outcomes. Adolescents who actively participate in structured sport activities in Asian school settings appear to have lower rates of school violence and disciplinary incidents (Higgins et al., 2020). This association may result from sport clubs providing an alternative social participation channel, redirecting energy toward constructive goals, and strengthening identification and commitment to the school community. In many communities in Latin America and Africa, where violence and social instability are major challenges, sport is given social functions beyond competition. Team sports such as football are often used to promote community peace, provide positive alternatives, and strengthen social cohesion, with observational studies suggesting positive community-level effects such as reductions in youth violence in local areas (Galinari and Bazon, 2021). However, many evaluations rely on observational data, and more rigorous studies are needed to determine causal effects.

Psychosocial outcomes. Research in Latin America has examined psychosocial risk profiles among adolescent offenders, revealing substantial heterogeneity. A latent-class analysis in Brazil identified distinct profiles of behavioral and psychosocial risk among adolescent offenders, implying the need for highly individualized interventions (Galinari and Bazon, 2021). Research in Chile similarly found that boys transitioning from the child welfare system to juvenile justice had particular combinations of risk factors, including weak family supervision, substance use, deviant peers, and risky leisure time (Zambrano et al., 2023). These findings suggest that sport interventions in these contexts must be tailored to address specific psychosocial risk profiles rather than adopting a one-size-fits-all approach.

Educational outcomes. Academic problems are closely associated with juvenile delinquency across Asian contexts. A study of adolescent offenders in Taiwan found that 89.76% had academic-achievement problems, and academic achievement was significantly associated with offense type and specific psychiatric disorders (Her et al., 2025). Integrating sport into school education may therefore not only improve fitness but also contribute to the prevention of school violence and delinquency by providing an alternative domain of success and belonging for academically struggling students. This educational protective function of sport is particularly relevant in East Asian contexts where academic pressure is high and school failure may serve as a pathway into delinquency.

Behavioral and health outcomes. Cross-cultural comparative research indicates that the success of exercise interventions depends on adaptation to local cultural contexts, social structures, and specific adolescent needs (Galinari and Bazon, 2021; Zambrano et al., 2023). Effective exercise interventions must be localized, taking into account socioeconomic conditions, education systems, gender norms, and community resources. Studies in North Africa have documented high prevalence of physical inactivity and sedentary behaviors among adolescents, with 74.2% of boys and 46.9% of girls being physically active, while a substantial proportion engaged in more than 2 h of daily sedentary screen time. These findings underscore the importance of understanding physical activity patterns in non-Western contexts and suggest that culturally adapted exercise interventions may be particularly relevant for regions where sedentary lifestyles and associated behavioral risks are prevalent.

Summary of evidence by outcome type. Across the three geographic regions, the evidence base reveals a consistent pattern: the strongest and most consistent evidence for exercise and sport interventions relates to delinquency-related outcomes, followed by psychosocial outcomes. Evidence for educational outcomes is more limited but suggestive of protective effects through enhanced school bonding. Evidence for behavioral and health outcomes beyond the immediate physical activity benefits remains the least developed, with substance use and sedentary behavior outcomes being inconsistently measured across studies. This pattern underscores the need for future research to adopt more comprehensive outcome assessment batteries that capture the full range of potential intervention effects, as well as longer follow-up periods to assess the durability of observed changes across all outcome domains.

## 6 Key moderators affecting intervention effects

### 6.1 Program design and implementation quality

The effectiveness of exercise training for juvenile delinquency depends largely on program design and implementation quality. Duration and intensity are critical factors determining whether interventions produce lasting effects. Short-term and sporadic programs are often insufficient to induce long-term behavioral or cognitive change, whereas longer and more frequent participation is associated with better outcomes (Meulen et al., 2026). A meta-analysis of Dutch juvenile justice interventions suggested that, for moderate- and high-risk youth, family-systemic interventions may be more effective when treatment lasts 6 months or longer, consistent with the risk-need-responsivity model (Meulen et al., 2026). This implies that exercise interventions must also involve long-term commitment. A structured program lasting at least one school year and held several times a week can provide adolescents with continuous opportunities to learn, practice, receive feedback, and build relationships, whereas one-off activities are unlikely to reshape identity or behavior.

The quality and role orientation of coaches are another core factor. In exercise-intervention programs, coaches are not merely instructors of sport skills but mentors and role models in adolescent development (McDonough and Knight, 2023). Coaches with knowledge of youth development, communication skills, and caring qualities can build trusting relationships with participants, and these relationships have therapeutic and guiding value. A national survey of sport programs in U. S. juvenile correctional facilities found that most institutions reported positive youth development as a purpose of their sport programs (McDonough and Knight, 2023). To achieve this goal, coaches must move beyond conventional athletic instruction and focus on personal growth, emotion management, and social-skill development. Their behavior, attitudes, and values subtly influence adolescents and help them build healthier self-concepts and relationships. Specialized coach training is therefore necessary, including trauma-informed care, basic counseling skills, and risk management.

The degree of structure in activities also requires careful balance. Completely free and unorganized play may not provide sufficient learning frameworks or boundaries, while highly structured and overly competitive training focused only on winning may increase frustration and aggression or reproduce negative pressures experienced elsewhere (McDonough and Knight, 2023). Effective design should balance structure and autonomy. Activities need clear rules, goals, and skill-development pathways, while also incorporating enjoyment, teamwork, and opportunities for personal challenge. Such balanced structured activities help adolescents learn rule compliance, deal with success and failure, and cooperate with others in a safe and predictable environment. Carefully designed sport situations allow adolescents to practice impulse control, emotion regulation, and conflict resolution, all of which are core life skills needed to reduce recidivism.

### 6.2 Individual characteristics of participants

Age and developmental stage are key factors influencing intervention effects. Preventive interventions for early adolescents aged 10–14 years may be more effective than corrective interventions for older adolescents with serious offending histories. This view is consistent with Moffitt’s developmental taxonomy, which distinguishes adolescence-limited from life-course-persistent offending trajectories (Ahonen et al., 2020). For youth whose behavior patterns are not yet consolidated, interventions have greater potential to shape prosocial behavior and character. The positive effects of the Only You Decide who you Are sport-based intervention suggest that intervention before risky behavior patterns become fully formed may be more effective (Meulen et al., 2026). In contrast, justice-involved older adolescents with repeated offending histories may require more intensive and integrated interventions.

Initial risk level is another core variable. Adolescents from high-risk environments, including poverty, family dysfunction, and childhood trauma, face multiple cumulative risks and may benefit most from comprehensive support integrating exercise with education and psychological services. Childhood trauma and abuse significantly increase the risk of juvenile delinquency (Alexander and Kerig, 2025). Trauma exposure, post-traumatic stress symptoms, dissociation, and reckless or self-destructive behavior are important mediating pathways to offending (Modrowski et al., 2021). For these high-risk youth, exercise alone may be insufficient to address complex psychosocial needs. Research in Chile found that youth transitioning from child welfare to juvenile justice systems were characterized by weak family supervision, substance use, deviant peer relationships, and risky leisure time (Zambrano et al., 2023). Exercise programs must therefore be combined with case management, psychological counseling, family support, and academic assistance.

**Figure 4:**
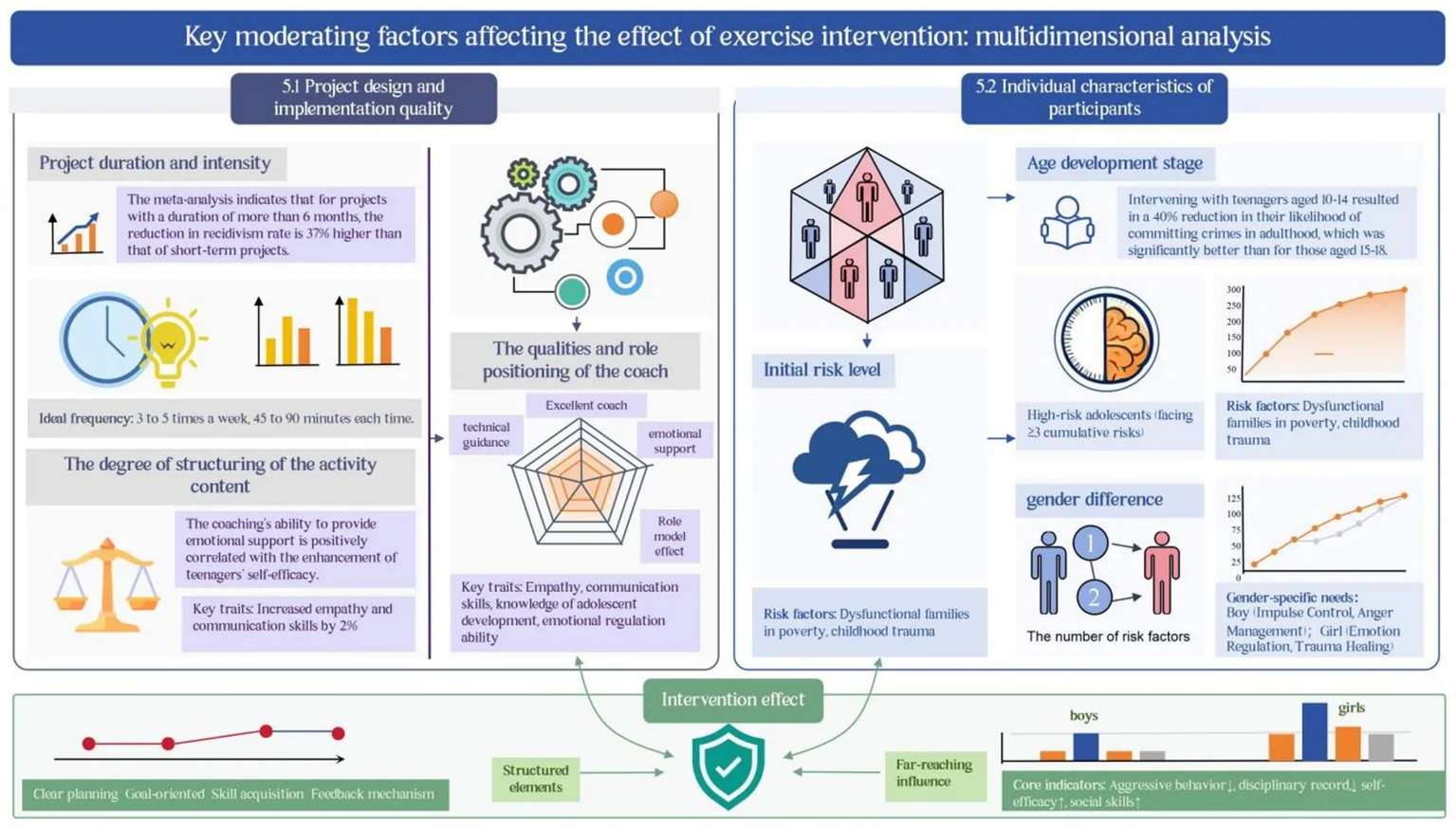
Key moderators of exercise-intervention effectiveness. After removal of the original figure, this figure has been renumbered. It summarizes how program design and implementation quality and participants’ individual characteristics interact to influence intervention outcomes.

Gender differences require gender-sensitive program design. Boys and girls differ not only in sport preferences but also in delinquency pathways, psychological characteristics, and responses to intervention. Male adolescent offenders are more numerous and often involved in violence and property crime (Galinari and Bazon, 2021). They may display more externalizing problems and greater association with deviant peers (Drozdova et al., 2022). Female offenders are fewer, but their risk factors may be more internalizing and related to emotional disorders, trauma exposure, and intimate-partner victimization (Harris et al., 2024). At the cognitive-affective level, male adolescent offenders show abnormalities in complex social emotions such as envy and schadenfreude, associated with reduced gray-matter volume in regions related to mentalizing and executive function (Franco-O’Byrne et al., 2021). Programs should therefore provide sport options and relationship-building strategies suited to different gendered experiences rather than assuming that a single model fits all participants (Figure 4).

## 7 Challenges and limitations of exercise intervention

### 7.1 Methodological challenges

Methodological challenges are central to evaluating the quality and reliability of evidence on exercise training for juvenile delinquency. First, random assignment is difficult. In community or justice settings, conducting strict randomized controlled trials with juvenile offenders involves both ethical and practical obstacles (Finch et al., 2021). Randomly assigning high-risk youth to intervention and control groups may raise fairness concerns, and the coercive nature of justice procedures, family willingness, and limited community resources make pure randomization difficult (Meulen et al., 2026). Many studies therefore rely on quasi-experimental designs, including non-randomized controlled trials and pre-post comparisons. Although these provide valuable observational data, they weaken causal inference and make it difficult to distinguish intervention effects from natural development, selection bias, or confounding factors.

Second, the lack of long-term follow-up data is a major limitation. Most studies focus on immediate or short-term effects, such as changes in behavior, attitudes, or physiological indicators during or shortly after intervention (Finch et al., 2021). However, the ultimate goal of intervention is to promote long-term social adaptation and character formation, reduce adult recidivism, and improve employment, education, and life outcomes. Systematic follow-up data on these outcomes remain scarce (Alexander and Kerig, 2025). Many studies assess aggression or social skills after intervention, but few follow participants into adulthood to examine criminal records, occupational stability, or overall life satisfaction (Ahonen et al., 2020). This ambiguity of endpoints makes it difficult to determine whether exercise interventions produce lasting positive transformation or merely temporary honeymoon effects.

Third, the reliability and validity of measurement tools are persistent problems, especially for constructs such as delinquent behavior and character. Delinquent behavior is often measured through self-report or official records, both of which have limitations (Finch et al., 2021). Self-report may be influenced by social desirability, with adolescents hiding or minimizing misconduct in sensitive justice contexts (Her et al., 2025). Official records such as arrest or conviction data may underestimate true delinquency because they miss undetected or unreported violations (Finch et al., 2021). Measurement error directly affects evaluation accuracy. For abstract constructs such as character, responsibility, empathy, and self-efficacy, there is no gold standard, and different studies use different tools, limiting comparability and synthesis. Future studies should combine multiple data sources, standardized scales, and objective behavioral indicators.

### 7.2 Barriers to practical dissemination

Although exercise-based interventions show potential for preventing and reducing juvenile delinquency, dissemination faces several structural barriers. Sustainable funding and resources are core challenges. Many effective programs rely on short-term government grants or philanthropic donations (Meulen et al., 2026). Unstable funding makes long-term operation difficult and limits both sustained effects and replicability. For interventions intended to shape character and behavior through long-term participation, intermittent funding can interrupt programs and deprive adolescents of continuous support. Professional shortages are another bottleneck. Effective programs require implementers to have not only sport expertise but also knowledge of adolescent psychology, trauma-informed practice, risk assessment, and interdisciplinary collaboration. Such personnel are often insufficient in community and justice settings.

A common misconception in practice is the myth of sport transfer, namely the assumption that sport participation automatically and inevitably leads to positive character development, such as self-discipline, cooperation, and respect for rules, and therefore reduces delinquency. This view ignores the need for intentional and structured design. Sport participation alone is insufficient to change deep risk factors such as weak family supervision, deviant peer association, substance use, or academic failure (Zambrano et al., 2023). If sport programs do not incorporate these risk factors into their intervention framework and do not include explicit components for teaching alternative thinking, empathy, and prosocial values, their effects will be limited. Sport may even reinforce aggression or exclusion if poorly supervised. Therefore, exercise intervention must be designed as an educational and developmental platform rather than merely as physical activity.

## 8 Synergistic integration of exercise intervention with other intervention modes

### 8.1 Integration with cognitive behavioral therapy

Combining core techniques of cognitive behavioral therapy (CBT) with exercise training is an innovative and effective integrated model for intervening in juvenile delinquency. The key is to use post-exercise circle talks or reflection sessions to introduce cognitive restructuring and problem-solving skills, helping adolescents identify and correct maladaptive thinking patterns that may lead to offending. Cognitive-behavioral physical activity has been shown to buffer the negative influence of rumination on mental wellbeing in adolescents (Derelioğlu et al., 2025), providing theoretical support for embedding CBT techniques into sport interventions. Sport itself can function as an ideal behavioral experiment. Conflicts and frustrations in team sports provide real situations in which adolescents can apply coping strategies learned in reflection sessions, such as using breathing instead of aggression. Because emotions are more vivid in sport contexts than in classroom discussion, cognitive-behavioral learning may be more easily internalized.

### 8.2 Integration with academic support and vocational training

Integrating exercise training with academic support and vocational training is a comprehensive strategy that addresses two key criminogenic risks: academic failure and economic hopelessness. Academic problems are closely associated with juvenile delinquency. A study of adolescent offenders in Taiwan found that 89.76% had academic-achievement problems, and academic achievement was significantly associated with offense type and specific psychiatric disorders (Her et al., 2025). Youth with lagging academic performance were more likely to commit property offenses, while dropouts showed more person-directed offenses (Her et al., 2025). This indicates that academic failure is not merely a developmental setback but a force that may push adolescents toward specific types of crime. A study based on youth offending services in Merseyside, United Kingdom, similarly highlighted educational alienation and truancy as pathways into offending (Filkin et al., 2022).

Academic support alone may not be sufficient because economic hopelessness is another deeper driver of delinquency. When adolescents cannot see future career prospects or economic independence because of school failure, they may be attracted by short-term illegal economic opportunities. Research in Chile identified socially maladjusted peer relationships and risky free time as key predictors of transition from child welfare to juvenile justice (Zambrano et al., 2023). Combining vocational training with sport programs can provide practical skills and future employment possibilities, directly reducing hopelessness. For example, programs may integrate sport participation with modules on coaching assistance, fitness-service skills, or community-event organization, enabling adolescents to transform sport interest into potential career pathways.

When designing mechanisms that use sport participation to motivate academic progress, caution is needed to avoid reducing sport to a purely instrumental reward. Sport has intrinsic value in promoting physical and mental health, team spirit, rule consciousness, and perseverance, all of which are protective factors against delinquency. If participation is treated merely as a bargaining chip for tutoring or punishment avoidance, adolescents may view sport as an externally imposed task, which may weaken intrinsic motivation and positive psychological experience. Situational action theory indicates that morality and self-control are central to offending propensity, and self-control is especially protective when strong moral values are present in high-risk environments (Guo, 2025). Exercise should therefore be integrated with academic and vocational support in a way that preserves its developmental meaning.

### 8.3 Integration with family intervention

Combining exercise training with family intervention is a key strategy for improving outcomes. The family is the central environment for adolescent development, and family functioning directly affects behavior. Family-level risks, such as parental deviant modeling, weak supervision, and dysfunction, are closely related to juvenile delinquency (van de Weijer and Kroese, 2025; Henning et al., 2025). For example, parental gambling significantly strengthens the likelihood that children with early deviant tendencies will engage in similar behavior, highlighting the influence of role models in the home (Walters, 2021). Interventions that include families by improving communication and parent–child relationships may strengthen protective factors at their source. A meta-analysis of Dutch juvenile delinquency interventions suggested that some traditional family-systemic interventions showed limited effects under certain conditions, but this does not negate the importance of family involvement; rather, it indicates that family support must be adapted to specific risk needs (Meulen et al., 2026).

**Figure 5:**
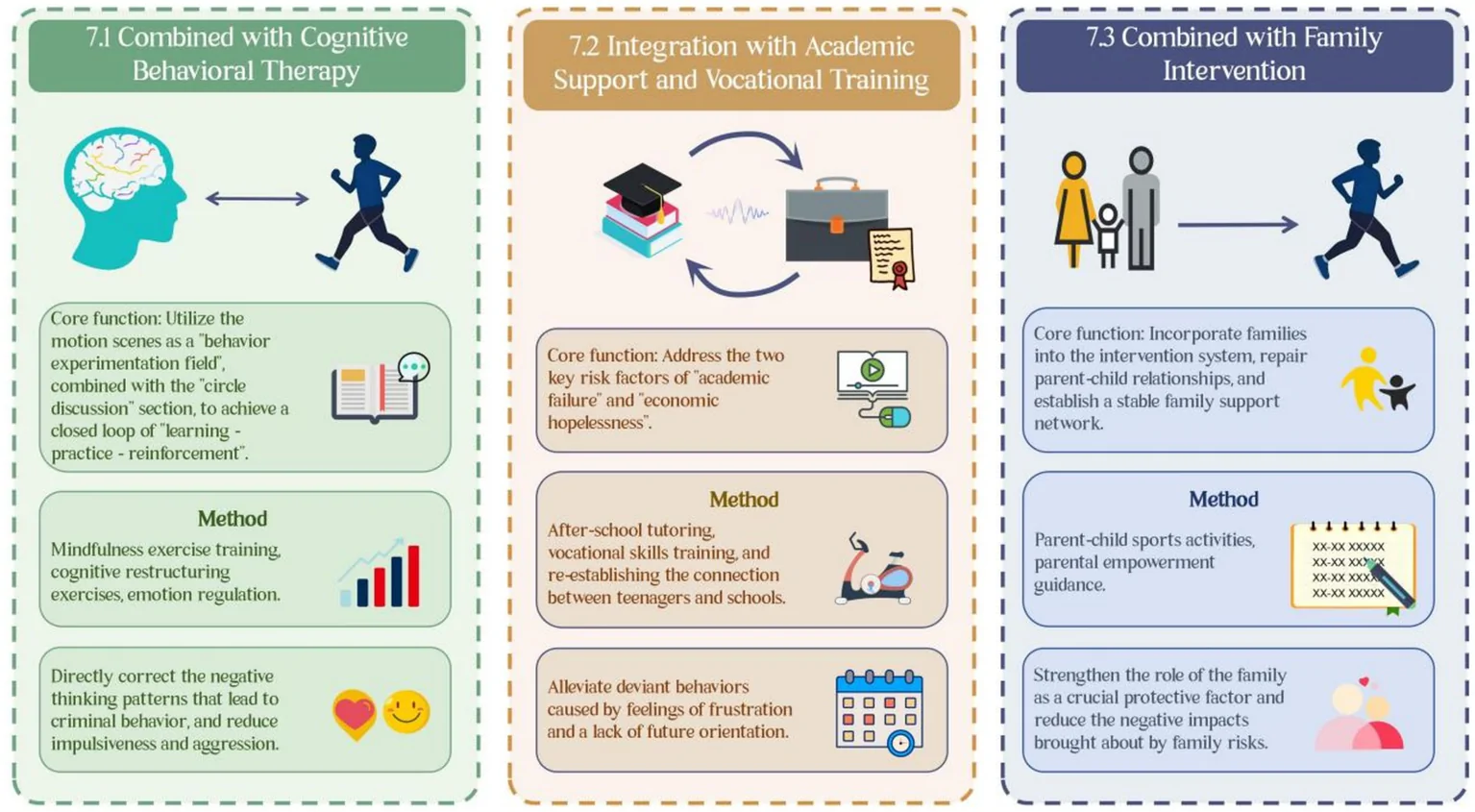
Integrated pathways of exercise intervention with other intervention modes. After removal of Figure 4, this figure has been renumbered. It shows how exercise intervention can be integrated with cognitive behavioral therapy, academic/vocational support, and family intervention to improve delinquency-prevention outcomes.

Providing systematic guidance and support for parents is another pillar of effective integration. Many parents understand the importance of supporting their children but lack concrete knowledge about how to use sport contexts for positive guidance. Programs should empower parents by teaching them how to support sport participation and use sport for character education. This includes encouraging training progress without excessive pressure, guiding children to interpret defeat constructively, and transferring values such as rule awareness, teamwork, and respect for opponents into family and social life. In interventions for children with ADHD, combining family-assisted training with staged comprehensive exercise interventions improved core symptoms and cognitive function, and family participation helped sustain training effects. For juvenile offenders, family involvement can similarly extend the influence of sport intervention beyond the field (Figure 5).

## 9 Policy implications and cross-sector collaboration

### 9.1 Integration of roles across justice, education, and social services

In the systematic prevention and intervention of juvenile delinquency, the integration of justice, education, and social-service roles is essential. These sectors need to move beyond traditional functional boundaries and treat developmental exercise programs as core intervention strategies. Justice departments may use participation in certified high-quality sport programs as a sentencing option or probation condition, providing structured positive alternatives for adolescents. A Dutch meta-analysis of interventions certified by the Netherlands Youth Institute found that the sport-based Only You Decide who you Are intervention showed effectiveness (Meulen et al., 2026). Incorporating structured physical activity into judicial decisions can provide positive role models, discipline training, and social-skill learning opportunities, replacing negative environments that may lead to reoffending. This integration focuses not only on punishment but also on character formation and social reintegration, consistent with the risk-need-responsivity model (Meulen et al., 2026).

Education departments play a foundational role and should view developmental physical education as an important component of whole-person development and risk prevention rather than merely as a curricular requirement. Juvenile delinquency is closely associated with academic achievement problems and psychiatric disorders (Her et al., 2025), and school attachment and commitment are significantly related to offending (Higgins et al., 2020). Education systems should therefore reposition physical education as a tool for promoting social–emotional learning, executive-function development, and positive peer relationships. High-quality physical education and after-school sport programs can help students develop positive school attachment, a key protective factor against delinquency (Higgins et al., 2020). Elements within sport, such as rule compliance, teamwork, coping with frustration, and goal setting, directly address risk factors common among juvenile offenders, including poor impulse control, social-cognitive deficits, and sensation seeking (Franco-O’Byrne et al., 2021; Loureiro et al., 2023).

Youth social-service agencies are key hubs connecting justice systems, education systems, and community resources. They can include sport referral and funding as part of case-management plans. When assessing risk and protective factors, social workers should treat organized sport participation as an important intervention resource. Risk factors for juvenile delinquency are multilayered, including executive-function deficits and emotion-regulation difficulties at the individual level, weak supervision at the family level, and deviant peers and risky leisure activities at the community level (Franco-O’Byrne et al., 2021; Zambrano et al., 2023). Through case management, social-service agencies can match adolescents with sport programs suitable for their interests and needs, such as team sports for peer-relationship improvement or individual sports for self-efficacy and future orientation. The Communities That Care program in Victoria, Australia, demonstrates the effectiveness of cross-sector collaboration in reducing youth crime at the community level (Rowland et al., 2022).

### 9.2 Funding investment and program certification standards

To ensure that exercise-training interventions for juvenile delinquency are scientific, effective, and socially sustainable, systematic funding support and quality-certification systems are crucial. Funding sources for programs targeting juvenile offenders or high-risk youth are often fragmented and unstable, directly affecting sustainability and evaluation rigor. An evidence and gap map of interventions for institutional child maltreatment found a substantial imbalance in the evidence base, with more than half of studies conducted in the United States and limited representation from countries with large populations and high rates of child maltreatment (Finch et al., 2021). Governments should therefore establish dedicated funds to support the development, implementation, and long-term evaluation of evidence-based exercise interventions. Priority should be given to programs that integrate multidisciplinary resources, target specific risks such as trauma exposure, substance use, and school alienation, and include clear evaluation frameworks (Modrowski et al., 2021).

Alongside funding, national or regional certification systems for program quality are needed to guide resources toward effective interventions. Such systems should specify standards for goals, design, staff qualifications, process monitoring, and outcome evaluation. Program goals should go beyond physical improvement and identify specific psychosocial risks to be addressed, such as increasing school attachment, reducing moral disengagement, strengthening emotion regulation, or interrupting deviant peer association (Higgins et al., 2020; Agudelo Rico et al., 2024). Program design should be based on established psychological or criminological theories, including situational action theory and the risk-need-responsivity model, and incorporate elements such as cognitive behavioral therapy and social–emotional learning (Guo, 2025). Staff qualifications should require coaches and counselors to possess sport expertise as well as basic training in adolescent development, trauma-informed care, and risk management (Finkelhor et al., 2025). Evaluation standards should require objective and multidimensional tools, measuring not only reductions in offending but also changes in intermediate variables such as self-efficacy, impulse control, and social–emotional competence, with long-term follow-up (Fernández-Moreno et al., 2024).

## 10 Future research directions

### 10.1 Deepening and refining mechanism research

Research on exercise training for juvenile delinquency is shifting from broad outcome evaluation to detailed exploration of internal mechanisms. The key is to use more precise tools and methods to reveal how exercise reduces delinquency risk by affecting specific psychological and physiological processes. Neuroimaging and physiological measurement offer unprecedented biological perspectives on how exercise may influence impulse control, empathy, and other processes closely related to offending. Juvenile offenders show abnormalities when experiencing complex social emotions such as envy and schadenfreude, associated with weaker executive functions and reduced gray-matter volume in brain regions involved in mentalizing and social–emotional processing (Franco-O’Byrne et al., 2021). Exercise may improve emotional regulation and social cognition by enhancing the function and structure of prefrontal-parietal executive-control networks. Research on attentional bias further indicates that adolescent offenders show bias toward threatening stimuli and faster detection of negative emotional targets, an atypical response pattern related to frontal-lobe function (Pino et al., 2023).

To clarify different pathways and their relative importance, researchers increasingly use longitudinal designs and advanced statistical models, such as growth models and mediation analysis. These methods can distinguish whether exercise prevents delinquency by enhancing self-efficacy, improving peer relationships, or changing other mediators. A study based on situational action theory and developmental ecological action models analyzed interactions among self-control, morality, and peer delinquency in 1,354 serious adolescent offenders (Guo, 2025). Lower offending was found when adolescents had stronger moral beliefs, higher self-control, and less deviant peer association. Self-control was most protective in high-risk environments when combined with strong moral values. This suggests that regular exercise may strengthen self-control through persistence and delayed gratification while promoting internalization of prosocial values. Studies of decision-making styles also show that rational decision-making relates to prosocial behavior through life satisfaction and positive emotions, while irrational styles such as hypervigilance relate to emotional and behavioral difficulties through negative emotions (Cardona-Isaza et al., 2025).

### 10.2 Cultural adaptation and global localization

Current research on exercise interventions for juvenile delinquency is concentrated mainly in Western cultural contexts, limiting applicability in non-Western settings. Studies of institutional child maltreatment show serious imbalance in research settings, with much evidence concentrated in schools or early-learning environments and little research in sport clubs, juvenile justice agencies, and other specific contexts (Finkelhor et al., 2025). A meta-analysis of juvenile delinquency prevention that included sport interventions was based mainly on Western judicial practices, such as those in the Netherlands, and its cross-cultural generalizability remains to be tested (Meulen et al., 2026). Future research should explore how sport type, coach style, and program goals can best fit different cultural contexts. In collectivist cultures with strong family ties, team sports may be more effective than individual sports in promoting social connection and prosocial value internalization. Coach leadership style should also be adapted to cultural differences, and program goals should align with local values, for example by integrating sport with educational support in cultures that emphasize academic achievement.

Combining local traditional sports and games with modern intervention theories is a key pathway for developing culturally resonant programs. Existing interventions are often based on Western psychological theories, such as CBT and positive youth development, and use mainstream modern sports, which may not match the everyday experiences and cultural identities of many non-Western youth. Traditional dance, martial arts, archery, and community games in Indigenous or minority communities often function not only as physical activity but also as carriers of culture, discipline, respect for elders, and community cooperation. Integrating these activities into interventions for delinquent youth can use their cultural meaning and emotional connection as natural media for character formation and social reintegration. Such development requires interdisciplinary cooperation among sport science, psychology, anthropology, and social work, as well as participatory action research with community leaders, coaches, adolescents, and families.

### 10.3 Integration of digital technology and exercise intervention

The integration of digital technology and exercise intervention provides new pathways for preventing and correcting juvenile delinquency. This field mainly involves wearable devices for precise monitoring and feedback and serious games or mobile applications that combine exercise challenges with prosocial-skill learning. Wearables such as smart bracelets and heart-rate monitors can track activity levels, heart rate, and sleep quality in real time, providing objective data for personalized adjustment of intervention plans. Immediate feedback can improve scientific rigor and increase interest and adherence through gamified designs such as daily step goals or exercise challenges. Digital remote monitoring and guidance can improve physical functions such as cardiopulmonary endurance and may indirectly influence behavior by enhancing self-efficacy. In juvenile justice settings, such technology can help staff monitor physiological states and participation levels and provide quantitative evidence for intervention evaluation.

Serious games and mobile applications that combine exercise challenges with prosocial skills are becoming important complements or entry points for offline programs. Virtual-reality or augmented-reality training can create immersive and interactive simulation environments. Adolescents can complete physically engaging tasks while learning conflict resolution, emotion management, and teamwork (Snow-Hill et al., 2021). A technology-based training tool for justice-involved youth showed that interactive modules teaching health promotion and social skills could improve knowledge and program engagement (Snow-Hill et al., 2021). Such digital therapies integrating physical activity with cognitive-behavioral training are especially suitable as supplements to face-to-face programs or as initial interventions in resource-limited settings (Piper et al., 2024). With artificial intelligence and big-data analysis, digital exercise interventions may enable more precise risk assessment and personalized program delivery.

## 11 Conclusion

This review synthesized international empirical evidence on exercise and sport-based interventions for juvenile delinquency, examining theoretical frameworks, mechanisms of action, program types, effectiveness, moderators, and implementation challenges. The review yields four core findings that are consistently supported by the available empirical evidence:

First, structured exercise and sport-based interventions can reduce delinquency-related outcomes, including aggressive behavior, disciplinary incidents, and recidivism, but the strength of this evidence is conditional. The most robust support comes from studies of well-designed, long-term programs with clear curricula and trained facilitators (Meulen et al., 2026), while short-term or unstructured activities show limited or inconsistent effects. The effect sizes vary considerably across studies, and the evidence for sustained post-intervention effects remains limited due to a lack of long-term follow-up data.

Second, the effectiveness of these interventions is critically dependent on program design and implementation quality rather than on exercise participation per se. Key determinants include program duration and intensity, coach qualifications and role orientation (as developmental mentors rather than merely skill instructors), and the degree of structured activity balanced with participant autonomy (McDonough and Knight, 2023; Meulen et al., 2026). These findings are supported by multiple studies across different geographic contexts, representing the most firmly established principle in this field.

Third, exercise interventions operate through three interconnected pathways—physiological (neurotransmitter regulation, prefrontal cortex development, stress-response modulation), psychological (self-efficacy enhancement, emotion regulation, impulse control), and social (positive adult mentorship, prosocial peer networks, rule internalization). However, the evidentiary support for these pathways is uneven: the social pathway has the strongest direct support from intervention studies (Mikytuck and Woolard, 2021; McDonough and Knight, 2023), the psychological pathway is supported by extensive correlational evidence but lacks direct mediation tests in intervention contexts (Cardona-Isaza et al., 2025; Loureiro et al., 2023), and the neurobiological pathway remains the most speculative, based on inferences from independent lines of research rather than direct tests within delinquency interventions (Franco-O’Byrne et al., 2021; Anderson et al., 2024).

Fourth, individual and contextual moderators significantly influence intervention outcomes. Age and developmental stage are consistently supported as moderators, with early adolescents (ages 10–14) showing greater responsiveness than older adolescents with established offending histories (Meulen et al., 2026). Gender differences in program access are well documented, with girls in juvenile justice settings consistently offered fewer sport opportunities than boys (McDonough and Knight, 2023). Family dysfunction, trauma history, socioeconomic disadvantage, and cultural context are identified as important moderators in observational research (Modrowski et al., 2021; Zambrano et al., 2023; Alexander and Kerig, 2025), although they have not been tested within sport intervention trials.

In conclusion, the current evidence supports the potential value of exercise and sport-based interventions as a developmental strategy for juvenile delinquency prevention and intervention. However, sport itself is not a universal remedy. Its positive effects depend on a carefully designed, theory-driven, and context-sensitive intervention ecology, and the evidence base remains methodologically heterogeneous and geographically concentrated. The ultimate goal is not merely to keep adolescents busy with physical activity, but to empower them through structured sport participation that strengthens their bodies while building character, competence, and positive social connections—thereby achieving mutual benefits for individual development and public safety. Achieving this goal will require sustained commitment from researchers, practitioners, and policymakers to generate more rigorous evidence, develop culturally adapted programs, and build cross-sector partnerships that support the healthy development of all young people, particularly those at greatest risk.
